# Relationship Between Adolescent Health Anxiety and Health-Related Internet Use: 3-Wave Longitudinal Survey Study

**DOI:** 10.2196/66129

**Published:** 2025-10-14

**Authors:** Adela Svestkova, David Smahel, Lenka Dedkova

**Affiliations:** 1 Interdisciplinary Research Team on Internet and Society Faculty of Social Studies Masaryk University Brno Czech Republic

**Keywords:** adolescence, adolescent, anxiety, Czech, health anxiety, health anxiety, health related internet use, health-related internet use, HRIU, hypochondriasis, internet, longitudinal study, longitudinal, random intercept cross-lagged panel model, RI-CLPM, well-being

## Abstract

**Background:**

Health anxiety among adolescents is understudied yet concerning. Health-related internet use (HRIU) is a common coping strategy, but disconcerting content may heighten, rather than mitigate, the health anxiety. While research has been conducted, within-person evidence for long-term fluctuations is lacking. Furthermore, adolescents with different base-level (initial) health anxiety may react differently to health-related content, making the effect dependent on health anxiety level compared to others.

**Objective:**

This study focused on the longitudinal relationship between HRIU and health anxiety on the within-person level in adolescents. We also considered their initial health anxiety, comparing adolescents with low, medium, and high base-level health anxiety.

**Methods:**

We analyzed data from 2500 Czech adolescents, aged 11-16 years (mean 13.43, SD 1.69 years; 1250/2500, 50% girls in Wave 1). Health anxiety was measured by the affective subscale of the Multidimensional Inventory of Hypochondriacal Traits, and HRIU was captured by 6 items reflecting health-related behaviors (eg, reading articles or watching videos with health-related content). The level of health anxiety in Wave 1 also served as a grouping factor. The data were collected in 3 waves, 6 months apart from June 2021 to June 2022, and analyzed using a random intercept cross-lagged panel model, chosen for its ability to separate within- and between-person effects.

**Results:**

On the between-person level, adolescents with higher base-level health anxiety were more frequent health-related internet users (β=.52; *P*<.001). On the within-person level, change in health anxiety did not predict change in HRIU, or vice versa, for those with high base-level health anxiety. In contrast, for adolescents with medium health anxiety, change in HRIU positively predicted health anxiety with large effects across all waves (β=.16; *P*=.03 from Wave 1 to Wave 2; β=.18; *P*=.01 from Wave 2 to Wave 3), and increase in health anxiety affected HRIU from Wave 2 to Wave 3 (β=.15; *P*=.03). For adolescents with low base-level health anxiety, change in their HRIU had large positive effect on changes in health anxiety from Wave 1 to Wave 2 (β=.17; *P*<.001), and health anxiety positively affected HRIU with medium effect from Wave 2 to Wave 3 (β=.11; *P*=.03).

**Conclusions:**

Adolescents with lower, rather than high, health anxiety are susceptible to the negative long-term effects of HRIU. For adolescents with high health anxiety, HRIU neither worsens nor relieves health anxiety over time, suggesting that counselors should recommend other coping strategies besides HRIU. Adolescents with medium to low health anxiety should be guided toward mindful HRIU to prevent increased health anxiety after HRIU. This includes fostering eHealth literacy (eg, recognizing personally irrelevant information and awareness of sensationalism in media) and coping mechanisms (eg, time or topic limits, or intentional rather than compulsive searching).

## Introduction

Since prepandemic times, evidence has shown a deterioration in adolescent mental health. Among other concerns, adolescents—characterized by heightened self-awareness and sensitivity to bodily changes—are particularly susceptible to health-related worries or rumination about health [1]. Yet, research has largely overlooked health anxiety—the fear of contracting or developing an illness—in this age group [2]. This gap is notable given that the COVID-19 pandemic likely intensified the decline [3] and that health concerns are central to any health crisis.

Health anxiety manifests through heightened attention to bodily sensations, an overestimation of the severity of minor symptoms, and the misinterpretation of benign signals as indicators of serious disease [1], resulting in increased health-related worries. While some vigilance can be adaptive, its excessive form leads to unnecessary precautions and social isolation and has comorbidities with other anxiety disorders [4,5]. Health anxiety can first develop in childhood or early adolescence [4,6], often triggered by observing a significant other’s serious illness or health-anxious cognitions [7]. During adolescence, rapid physical changes associated with puberty make it harder to distinguish normal bodily sensations from signs of illness [8]. This uncertainty can foster bodily hypervigilance, increasing the risk of misinterpreting sensations as serious symptoms [1]. Because health anxiety often persists into adulthood and frequently co-occurs with other anxiety disorders, addressing it during adolescence is particularly important [5,6].

Health anxiety is closely tied to health-related internet use (HRIU), a resource accessed by up to 80% of adolescents [9,10]. While illness-related information is a common target—whether concerning themselves or their relatives [9,11]—adolescents also turn to the internet for developmental and sensitive topics, such as fitness, body image, and sexual or mental health [12]. For some, online search serves as an alternative to consultations, motivated by fear of rejection, reluctance to trouble parents or doctors, or a desire for privacy—concerns especially salient during adolescence [13,14].

This association can be approached from two perspectives. The cognitive-behavioral model [15] suggests that people with higher health anxiety are consistently and differentially susceptible to reassurance seeking as a coping strategy [14]. At the between-person level, this is evident; individuals with higher health anxiety tend to engage more users, a pattern confirmed by meta-analytic evidence from 44 studies in adults [16]. Despite the general lack of literature on adolescent health anxiety, this pattern is consistently documented in adolescents as well [11,13]. Yet, the nature of this link may be more complex than a simple cause-and-effect relationship, as shown by an alternative theoretical approach by Brown et al [17]. Their integrative model shifts the focus to a within-person process that may apply broadly to HRIU, regardless of one’s level of health anxiety compared to others. Here, each search can generate both reassuring and worrying outcomes, which can either terminate or sustain further searching. In this way, HRIU can simultaneously ease and exacerbate health anxiety, fueling a vicious cycle [17]—an effect likely to be particularly pronounced during pandemic times [18].

While complementary, the two theoretical models are somewhat in tension. While the integrative model posits that its mechanisms should be comparably valid for all HRIU [17], the cognitive-behavioral model suggests differences for people with different base-level health anxiety [15]. As a result, the literature remains inconclusive on whether health anxiety and HRIU co-occur or causally affect each other, whether such effects may persist over months, and whether and how people with different base-level health anxiety are differentially susceptible to these mechanisms. Finally, literature on this topic has generally neglected adolescents, leading us to derive most of our assumptions from research conducted on adults. This study aims to fill these gaps by integrating two perspectives. In line with the integrative model [17], we will study how within-person fluctuations in health anxiety and internet use affect each other over time. Further, to account for the differential susceptibility outlined in the cognitive-behavioral model [15], we will separately study adolescents with different base levels of health anxiety, with a novel focus on the understudied group with lower health anxiety.

Regardless of their baseline level of health anxiety, adolescents may experience temporary spikes in anxiety due to factors such as illness in the family, changes in health status, or broader societal issues, like the COVID-19 pandemic [19]. In line with the adaptive nature of health anxiety, such increases can prompt information seeking, safety precautions, or help seeking [4,18,19], often leading to momentarily higher HRIU [17]. However, the integrative model suggests that these temporary increases may develop into lasting changes in internet use that persist beyond the triggering situation. Reassuring outcomes can reinforce positive beliefs about the internet as a valuable source of health information and relief from anxiety, whereas worrying outcomes can prolong the search in pursuit of reassurance or for compulsive reasons [17,18].

Simultaneously, in multiple stages of the process, HRIU can, in turn, impact health anxiety [17]. First, information from the internet may serve as the initial trigger of health-related worries [20,21]. Second, the prolonged increase in HRIU outlined in our first hypothesis may also contribute to an increase in health anxiety due to feelings of uncertainty and information overload [14]. Over time, this is expected to cause long-term health-related worries [19], potentially increasing health anxiety to a problematic level, although this assumption remains rather theoretical and untested [17]. Consistent with previous findings, we expect adolescents with higher base-level health anxiety to use health-related internet resources more frequently. Simultaneously, we anticipate that an intraindividual increase in health anxiety will lead to increased intraindividual HRIU 6 months later, and vice versa:

Hypothesis 1 (H1): Within-person changes in health anxiety will be positively associated with within-person changes in HRIU 6 months later.

Hypothesis 2 (H2): Within-person changes in HRIU will be positively associated with within-person changes in health anxiety 6 months later.

At the same time, an individual’s baseline level of health anxiety tends to remain relatively stable compared to peers [19], with higher levels being linked to specific cognitive patterns. These include more catastrophic cognitions, which explain increased worries and misinterpretation of sensations [15], and greater intolerance of uncertainty [22]. This increases their need for reassurance [21,23], including HRIU [14]. As a result, different base-level health anxiety seems to modify HRIU, leading to different risks of the vicious cycle of health worries and search in a systematic way [17,24].

On the one hand, adolescents with higher health anxiety are less likely to find reassurance, terminate the search, and return to their average usage levels [25]. As a result, they should be more likely to respond to a temporary increase in health anxiety with an internet use pattern observable over time. This should be amplified by their cognitive strategies [14]. In studies where they could engage with information of their choice, adolescents with higher base-level health anxiety explored topics more prone to escalation [26,27]. In a unified task, they were more likely than others to ignore probabilities of distressing information due to catastrophic cognitions, leading to more distress from the same content [24]. As a result, adolescents with higher base-level health anxiety may be more at risk of the vicious cycle.

On the other hand, this assumed trend may not be infinite. A sole longitudinal study by te Poel et al [28] examined the intraindividual relationship between health anxiety and HRIU over the period of months. The study, conducted on adults with 2-month intervals, analyzed participants with high baseline health anxiety separately from those with lower levels. Contrary to theoretical assumptions, no long-term relationship between health anxiety and internet use was detected among individuals with high baseline health anxiety, whereas a bidirectional positive effect was observed in those with lower levels. These findings suggest a ceiling effect, in which persistently high health anxiety and internet use show little within-person variability, rendering them no longer responsive to each other [28]. This is important, as it shows that people with high base-level health anxiety may not be as susceptible to the vicious cycle as expected, if at all.

However, we do not know the level of health anxiety where these 2 trends meet. Te Poel et al [28] compared adults with high health anxiety to all others; that is, they combined people with moderate and low levels together. Yet, adolescents with low health anxiety further differ in their HRIU and associated worries. A cross-sectional study showed that those with medium health anxiety resemble those with high health anxiety rather than those with low anxiety in terms of their internet use and COVID-related anxiety [13]. This marks the importance of a more nuanced focus on adolescents with different base levels of health anxiety.

To fill this gap, we aim to focus on these 2 contradictory, yet likely mutual, perspectives. In line with the theory, we expect that comparatively higher health anxiety might predispose individuals to a more pronounced within-person relationship between health anxiety and HRIU [20,23,24]. At the same time, both variables may reach their plateau at a certain level and will not affect each other anymore due to limited variance, contrary to the cognitive-behavioral model [28]. To address this discrepancy, we will examine these bidirectional within-person links separately for adolescents with high, medium, and low baseline anxiety, in line with the following research questions (RQs):

RQ1: Will the effect of change in health anxiety on change in HRIU be present in adolescents with high, medium, and low base levels of health anxiety compared to their peers?

RQ2: Will the effect of change in HRIU on change in health anxiety be present in adolescents with high, medium, and low base levels of health anxiety compared to their peers?

This study aims to provide new insights into intraindividual fluctuations in health anxiety and HRIU among adolescents with different base-level health anxiety. First, in line with the integrative model [17], we expect that health anxiety and HRIU will not only co-occur but also causally affect each other over time. Second, in line with the cognitive-behavioral model [15], we assume that this relationship may be modified with a growing base level of health anxiety. At the same time, we aim to replicate the findings from te Poel et al [28], which are somewhat contrary to the cognitive-behavioral model, suggesting that this growth may not be infinite. We will study whether the ceiling effect detected in adults is also present in adolescents. To provide better insight into who may be at risk and detect potential differences, we will focus on adolescents with low and medium base levels of health anxiety separately.

To test our assumptions, longitudinal insight into this difference is needed. While emerging evidence shows that health anxiety and HRIU also co-occur in adolescents [11,13], their long-term relationship may, but does not have to, be comparable to adults. As noted above, adolescents may differ from adults in the health information they seek, in rumination over it, or in recovery from distress over time [13,29]. Therefore, it is important to know whether increased HRIU leads to an increase in health anxiety detectable over months [17], suggesting a detrimental effect of HRIU on mental well-being.

In this study, we will examine the effects over a notably longer period compared to te Poel et al [28], analyzing data from 3 waves, each 6 months apart, providing the longest study of health anxiety and HRIU so far. This time frame allows us to study effects stable across events, such as short-term health issues and the COVID-19 pandemic, and see whether the observed longitudinal effects are able to hold over a longer period of time, suggesting a persistent change in health anxiety.

**Figure 1:**
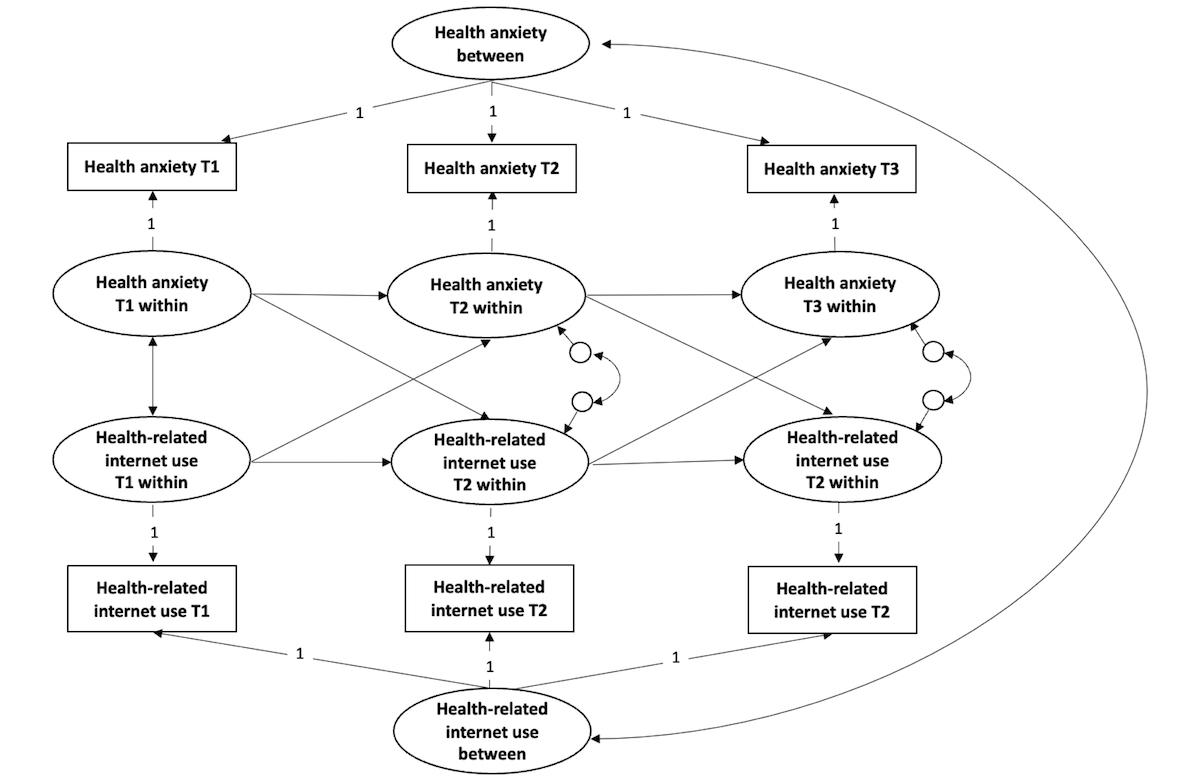
Conceptual model of the hypothesized longitudinal relationship between health anxiety and health-related internet use in Czech adolescents. T1: Wave1; T2: Wave 2; T3: Wave 3.

Figure 1 summarizes the conceptual model of the proposed interactions between health anxiety and HRIU over time. The effect hypothesized in H1 is represented by the cross-lagged paths from health anxiety in Wave 1 to HRIU in Wave 2 and the same path from Wave 2 to Wave 3. The effect described in H2 represents the opposite direction (ie, from HRIU to health anxiety in the subsequent wave).

## Methods

### Recruitment

We analyzed longitudinal data from 2500 adolescents, aged 11-16 years (Wave 1), collected through an online survey as part of a broader study on well-being and health in Czech adolescents within the DigiWELL Project (Research of Excellence on Digital Technologies and Wellbeing; CZ.02.01.01/00/22_008/0004583), which is cofinanced by the European Union, at Masaryk University. To include adolescents with various levels of health anxiety, we collected data from a community sample representative of the general Czech adolescent population. The age range was chosen to include the entire period of adolescence (ie, roughly 11-18 years), while excluding adolescents aged 10 or 17 years from Wave 1. This ensures that the pubertal transition had started before the data collection. Moreover, previous research has shown that there is a continuity of health anxiety from age 11 to 16 years [4]. From the perspective of the variables of interest, the Czech Republic is a country with high technology provision among adolescents, comparable to (or slightly higher than) similar European countries [30], and with an advanced health care system, again comparable to similar European countries, and with accessible health care guaranteed by the law.

The data collection was conducted in 3 waves, 6 months apart: June 2021 (Wave 1), November-December 2021 (Wave 2), and June 2022 (Wave 3). Eligibility was limited to Czech adults and their adolescent offspring within the requested age range (11-16 years in Wave 1). To ensure representation of Czech households with children, quotas were set for region (based on Nomenclature of Units for Territorial Statistics 3), municipality size, and the education of the head of the family. As this study was a part of a broader survey covering various topics, municipality size and region were chosen to represent the diversity and structural differences of the population, as has been done in previous projects [31]. In the area of health, these differences impact factors such as education (including health literacy), health care accessibility, health beliefs, and pandemic and prepandemic mortality rates [32]. Furthermore, we required equal representation of adolescents’ gender and each age group (by year). In Wave 1, a total of 2500 adolescents (mean age 13.43, SD 1.69 years; 1250/2500, 50% girls) completed the questionnaire. Of these, 1654 (794/1654, 48% girls) participated in Wave 2 and 1102 (529/1102, 48% girls) in Wave 3.

The data were collected by a professional research agency specialized in data collection, which applies strict quality checks and follows ethical standards and guidelines of the European Society for Opinion and Market Research. This approach was chosen as such agencies have access to a wide pool of participants and specialize in ways to prevent attrition during the study. The agency recruited participants from its online panel (approximately 165,000 panelists willing to participate in surveys), using procedures unknown to the authors. The aim was to obtain 2500 participating households completing the questionnaire in sufficient quality, which also determined the number of participants with specified sociodemographic characteristics related to the quota (eg, after reaching 1250 valid questionnaires from girls, households with girls would not be eligible anymore). Participants were invited through email and filled out the survey on the internet using the computer-assisted web interviewing method. The agency approached 17,502 panelists, 8287 of whom opened the questionnaire, 6818 were eligible based on the set quota, and 3124 of them completed the questionnaire. After data quality checks, 624 (20%) surveys were discarded, based on an excessive amount of missing data or low quality of answers (logical inconsistency or unrealistically short time spent with the questionnaire). This resulted in 2500 participants in Wave 1 who were eligible and completed the questionnaire in full and in sufficient quality. The sample size was determined with regard to the minimal criteria necessary for the intended analyses in the project. The sample size in Wave 3 was supposed to exceed 1000 adolescent-caregiver dyads to allow for multigroup random intercept cross-lagged panel model (RI-CLPM) analyses, and the attrition in each wave was not to exceed 34%.

Of the original 2500 participants, 1654 (33.8% attrition) also took part in Wave 2, and 1102 (33.3% attrition from Wave 2 to Wave 3) completed all 3 waves. Participants who decided to quit did not differ from those who completed the study in terms of adolescents’ age (*F*_2, 2497_=1.98; *P*=.14), health anxiety (*F*_2, 2497_=0.521; *P*=.59), HRIU (*F*_2, 2492_=0.253; *P*=.78), or gender representation (*χ*^2^_2_=5.52; *P*=.07), suggesting no severe attrition bias in the data. Therefore, we assume that the data are missing at random, which allows us to use full information maximum likelihood. Item nonresponse ranged from 0% to 1% for health anxiety items and below 0.1% for HRIU items across the waves.

### Procedure

Before the main study, the survey was pretested through cognitive interviews with 30 adolescents and 2 parents, followed by a pilot study with 195 adolescent-caregiver dyads to ensure the clarity of the questions, review data distributions, and verify the dimensionality and reliability of the scales.

A computer-assisted web interviewing method was used. All participants and their caregivers provided their informed consent over the internet when completing the questionnaire. For the adolescent questionnaire, both the caregiver and the adolescent provided their consent. The survey began with a short introduction, informing adolescents that each item included the options “I don’t know” and “I prefer not to answer.” The caregivers filled out the survey first and then handed it over to the adolescents. Parents were also able to view a PDF version of the adolescent questionnaire before consenting to their offspring’s participation. Before letting the child fill out the questionnaire, the caregiver was asked to provide the child’s demographic characteristics to ensure that the same child participated in all waves. Upon completing the questionnaire, participants were debriefed and reminded that by clicking “Continue,” their responses would be locked and inaccessible to their parents.

### COVID-19 Context

This study was designed shortly before the COVID-19 pandemic, and its evolving nature made it impossible to incorporate COVID-19–related variables in the study. However, we acknowledge retrospectively that the COVID-19 pandemic evolved dynamically throughout the data collection, potentially interfering systematically with our variables. During the pandemic, the restrictions were the same for the whole country. Wave 1 data collection was conducted during the calmest phase since the declaration of the pandemic, with lower incidence rates and nearly no restrictions. However, it immediately followed the peak of the pandemic, characterized by the highest incidence and death rates, as well as severe restrictions, including movement limitations. As a result, this period likely contributed to substantial variance in health anxiety and HRIU, reflecting personal experiences and the individual salience of COVID-19.

During Wave 2, the situation worsened, reaching the highest infection numbers since the declaration of the pandemic, accompanied by strict measures, such as limited access to services based on noninfectivity or vaccination status. The number of infections, as well as the severity of restrictions, was comparable across the country. As a result, we can expect an increase in health anxiety and intensified HRIU, but at the same time assume that the situation was relatively comparable throughout our sample.

By the time of Wave 3 data collection, the number of infections had decreased and remained consistently low. Most remaining restrictions had been lifted (with exceptions for social care and medical facilities), and prepandemic rules were largely restored [33]. Similar to Wave 2, we can assume that the situation was comparable for most participants, this time likely not contributing to either acute health anxiety or responsive HRIU much.

### Measures

#### Health Anxiety

Health anxiety was assessed using 4 items, which represent the affective subscale of the Multidimensional Inventory of Hypochondriacal Traits [34]. Participants indicated how strongly they agreed with the following: “I worry a lot about my health,” “When I experience pain, I fear I may be ill,” “Reading articles about disease makes me worry about my health,” and “I am concerned with the possibility of being diagnosed with a serious disease.” Responses were on a scale from 1 (very untrue) to 5 (very true). The scale demonstrated good reliability (0.85-0.89 across waves) and high item-rest correlations (*r*>0.65). Results supported full metric and partial scalar multigroup invariance (Δ*χ*^2^_13_=21.40; *P*=.65) and partial metric longitudinal invariance (Δ*χ*^2^_7_=9.36; *P*=.23). With regard to Steinmetz [35], we decided to approach the scale as a scalar noninvariant, comparing only the latent mean between the groups, as is possible for metric invariant scales, but not the factor loadings of individual items. Accordingly, we further handle health anxiety as the mean value of the items. On the other hand, reaching only partial metric longitudinal invariance should not affect the mean differences of the latent mean significantly [35], allowing us to approach the scale as longitudinally metric invariant and compare the mean values over time. Mean values of health anxiety with SDs for the whole sample as well as the individual groups across all waves are presented in Table 1.

**Table 1:** Means and SDs for health anxiety and health-related internet use (HRIU) in Czech adolescents aged 11-16 years at baseline (N=2500; 3-wave longitudinal study with 6-month intervals; data collected from June 2021 to June 2022).

		Wave 1, mean (SD)		Wave 2, mean (SD)		Wave 3, mean (SD)
**Full sample**						
	Health anxiety	2.47 (0.98)	2.50 (0.99)		2.42 (0.97)	
	HRIU	1.94 (1.09)	2.06 (1.18)		1.93 (1.11)	
**Low health anxiety in Wave 1**						
	Health anxiety	1.55 (0.37)	1.98 (0.85)		1.93 (0.79)	
	HRIU	1.61 (0.83)	1.76 (0.94)		1.66 (0.93)	
**Medium health anxiety in Wave 1**						
	Health anxiety	2.48 (0.91)	2.45 (0.78)		2.41 (0.84)	
	HRIU	1.92 (1.01)	2.03 (1.13)		1.90 (1.05)	
**High health anxiety in Wave 1**						
	Health anxiety	3.56 (0.57)	3.14 (0.91)		3.02 (0.91)	
	HRIU	2.34 (1.27)	2.43 (1.35)		2.28 (1.25)	

#### Initial Level of Health Anxiety as a Grouping Variable

The level of health anxiety measured in Wave 1 was used to group participants into approximately even thirds, representing low, medium, and high levels of adolescents’ health anxiety compared to their peers. This multigroup approach was chosen to meet analysis requirements and align with previous studies [13,28], aiming to highlight potential contrasts rather than establishing strict cutoff points. The cutoffs were selected to ensure adequate group sizes for analysis. However, due to ties in percentiles, the groups are not perfectly balanced (n=886 for high, n=561 for medium, and n=1053 for low health anxiety). Despite this, the grouping provided relative balance compared to methods such as splitting by SDs (Table 1).

#### Health-Related Internet Use

HRIU was assessed using a 6-item scale for general internet and social media use [36]. The scale had previously been adapted for health-related purposes by adding “regarding health, or illness, including COVID-19” to the instruction [13]. Participants responded to the instruction, “On the internet, one can find various contents (photos, videos, texts) regarding health or illness, including COVID-19. How often do you engage in following behaviors?” on a scale from 1 (never) to 7 (several times a day). The behaviors included “reading articles and posts with such content,” “reading discussions with such content,” “watching videos or viewing pictures with such content,” “replying to such content, eg, liking or voting,” “sharing others’ content, eg, through Instagram stories or on Facebook,” and “commenting on such content.” The items represented various ways of interacting with online health-related content and were focused more on the salience of health-related content than on specific topics or motivations, which is in line with the theoretical framing of this study.

Mean values of HRIU with SDs for the whole sample as well as the individual groups across all waves are presented in Table 1. We consider HRIU as a formative rather than reflective construct, in accordance with the criteria established by Hanafiah [37]. Crucially, health-related behaviors we assessed constitute, rather than reflect, HRIU. HRIU is multifaceted, and various combinations of behaviors can meaningfully lead to the same construct level, while each contributes in a unique, noninterchangeable way (eg, a person can be an intensive health-related internet user and yet never comment on health-related content). As a result—and unlike in reflective constructs—these behaviors may, but do not have to, correlate, preventing a meaningful reliability assessment. Therefore, we did not compute reliability or measurement invariance for this construct. This conceptualization is important to ensure that the behavior may, but does not have to, be linked to health anxiety and health-related worries (eg, compared to cyberchondria, which is now perceived as a topic-specific type of excessive internet use).

### Statistical Analysis

We used R software (version 4.2.2; R Foundation for Statistical Computing) to assess measurement invariance for health anxiety across groups and over time. The primary analysis was performed in Mplus (version 8.10; Muthén & Muthén) using the syntax for the multiple group RI-CLPM provided by Mulder and Hamaker [38]. The RI-CLPM enabled us to examine the causal relationship between within-person fluctuations while still acknowledging stable between-person differences, which is not possible with the cross-lagged panel model [39]. This distinction was crucial for assessing whether changes in one variable predicted subsequent changes in another within individuals rather than reflecting stable between-person differences, which is a key assumption theoretically framing our study, as we outlined in the Introduction*.* We did not consider other possible techniques, such as the latent curve model with structured residuals, as we did not expect systematic change in our variables over time.

To address missing data from item nonresponse and attrition, we used full information maximum likelihood, which was preferred over other methods, such as multiple imputation, as it uses all available data. The model was estimated using the robust maximum likelihood estimator, which accounts for nonnormality by providing robust SEs and scaled chi-square tests without introducing additional randomness [40]. Both variables were treated as manifest and computed as the means of the relevant items. We did not enforce time constraints, as the variables could vary across waves, for example, due to the COVID-19 pandemic. Due to the complexity relative to our sample size, the multigroup analysis could not be computed. Therefore, to answer RQ1 and RQ2, we computed the analysis separately for each group and did not compute multigroup differences. Model fit was evaluated based on the criteria established by Hu and Bentler [41]. The effect sizes were interpreted in line with the guidelines from Orth et al [42] for cross-lagged effects (β=.03 for small, β=.07 for medium, and β=.12 for large effects) and using a 5% significance level. The data and analysis scripts are available through the Open Science Framework [43].

### Ethical Considerations

The procedure and content of the questionnaire were approved by the Research Ethics Committee at Masaryk University (EKV-2018-068). The incentive for filling out each questionnaire was determined by internal rules of the agency and is unknown to the researchers but represented 160% of their standard incentive based on the questionnaire length to encourage the respondents to participate in the longitudinal design.

Given the COVID-19 context, the questionnaires were both filled out over the internet on one device and most likely from home. Parents were asked not to be present for the adolescent part of the questionnaire to ensure the child’s privacy. We then asked participants whether an adult watched or interfered during the survey. While a vast majority of caregivers seemed to comply with this rule, no other enforcement mechanisms could be applied due to the study’s nature. To compensate for potential sensitivity of some scales, we provided contacts related to mental health support, emergency, and guidelines for healthier internet use at the end of each questionnaire.

## Results

The data analysis proceeded in 2 steps. First, we conducted an RI-CLPM analysis on the full sample without grouping. According to the criteria established in Hu and Bentler [41], this model fit the data well (*χ*²_1_=0.159; *P*=.69; root-mean-square error of approximation [RMSEA]=0.00, 90% CI 0.00-0.04; comparative fit index [CFI]=1; Tucker-Lewis index [TLI]=1; standardized root-mean-square residual [SRMR]=0.002). Next, we introduced health anxiety level as a grouping factor (with 3 categories: low, medium, and high) and performed a multigroup RI-CLPM analysis (Table S1 in Multimedia Appendix 1). This model also demonstrated a good fit (*χ*²_3_=3.899; *P*=.27; RMSEA=0.02, 90% CI 0.00-0.06; CFI=1; TLI=0.99; SRMR=0.01).

**Figure 2:**
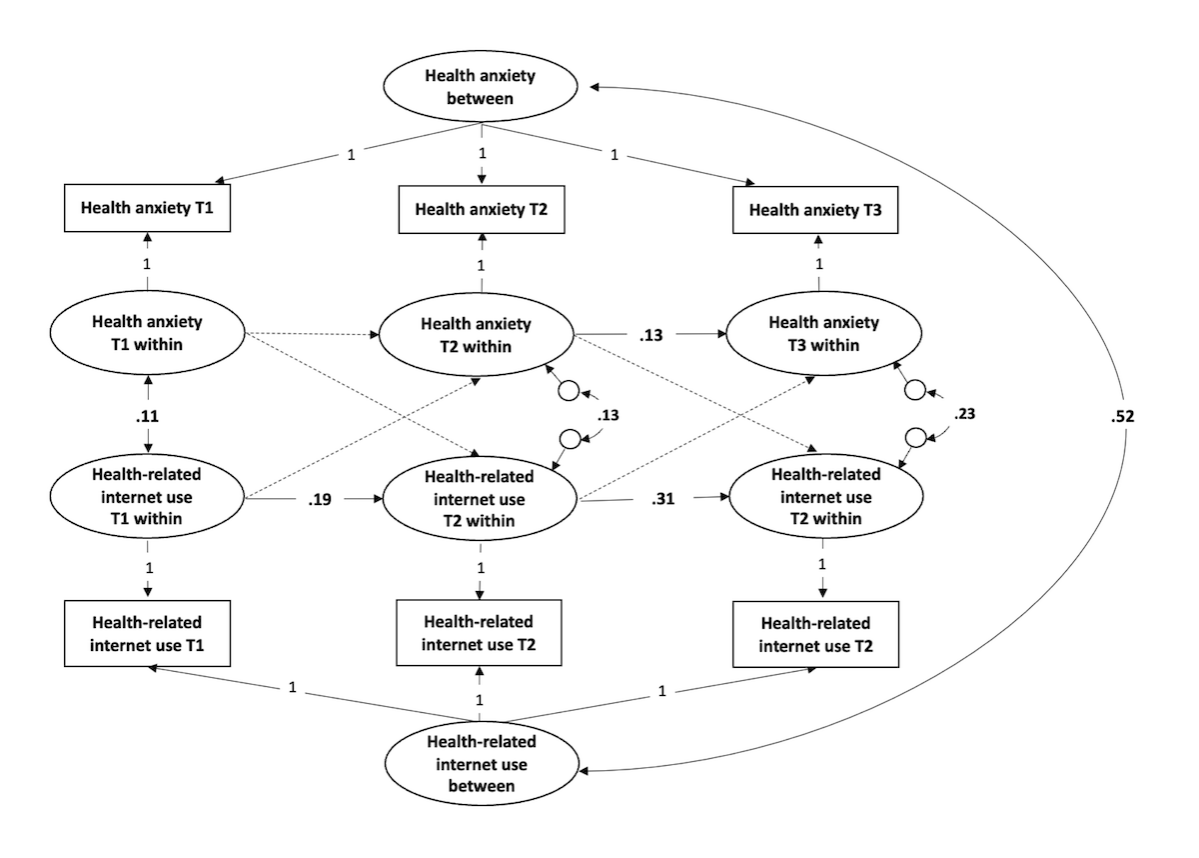
Results of the ungrouped model testing the longitudinal interaction between health anxiety and health-related internet use in Czech adolescents aged 11-16 years at baseline (3-wave study with 6-month intervals; data collected from June 2021 to June 2022; N=2500). For better orientation, only results that are significant at the 5% level are included. The effects that are nonsignificant at the 5% significance level are marked using a dotted line. For actual *P* values, see Table S1 in Multimedia Appendix 1. T1: Wave1; T2: Wave 2; T3: Wave 3.

Figure 2 presents a summary of the results for the model without grouping. On the between-person and cross-sectional levels, health anxiety and HRIU were strongly positively correlated (β=.52; *P*<.001), indicating that adolescents with higher health anxiety were more frequent users of health-related internet resources. A similar, albeit weaker, effect was observed in the subgroup of adolescents with comparatively high health anxiety (β=.18; *P*=.048), while no correlation between health anxiety and HRIU was revealed when studying only the subgroup with comparatively medium health anxiety or comparatively low health anxiety.

In H1, we assumed that the within-person change in health anxiety would predict a change in HRIU 6 months later. However, in the ungrouped model, we did not find support for H1, concluding that a change in health anxiety does not lead to a change in HRIU. H2 tested the other direction of the relationship, that is, a change in HRIU will lead to a change in health anxiety 6 months later. In partial support for H2, we found that change in HRIU in Wave 2 positively predicts change in health anxiety in Wave 3, with a medium effect size (β=.10; *P*=.048).

To get better insight into the process, we tested the same assumptions separately for adolescents with comparatively high, medium, and low health anxiety (RQ1 and RQ2). RQ1, similar to H1, asked whether the change in health anxiety leads to a change in later HRIU in adolescents with different base levels of health anxiety. For adolescents with comparatively high health anxiety, change in health anxiety did not predict change in HRIU, similar to the ungrouped results. On the other hand, a change in health anxiety in Wave 2 positively predicted change in HRIU in Wave 3 with a large effect size for adolescents with medium health anxiety (β=.15; *P*=.03) and with a medium effect size for those with low health anxiety (β=.11; *P*=.03).

To sum up, we can conclude that even after inspecting adolescents with high, medium, and low base-level health anxiety separately, we did not find any effect of change in health anxiety in Wave 1 on HRIU in Wave 2. On the other hand, we detected this effect from Wave 2 to Wave 3, but only for adolescents with medium to low base-level health anxiety.

In RQ2, we asked about the other direction of the effect, that is, whether a change in HRIUs leads to changes in health anxiety later for adolescents with comparatively high, medium, and low levels of health anxiety. Again, a change in HRIU did not predict a change in health anxiety for adolescents with comparatively high health anxiety. On the other hand, change in HRIU positively predicted change in health anxiety for adolescents with comparatively medium health anxiety, with large effect sizes both from Wave 1 to Wave 2 (β=.16; *P*=.03) and from Wave 2 to Wave 3 (β=.18; *P*=.01). For adolescents with low base-level health anxiety, change in HRIU in Wave 1 positively predicted change in health anxiety in Wave 2 with a large effect size (β=.17; *P*<.001) but no such effect was present from Wave 2 to Wave 3.

To conclude, according to the multigroup model, a change in HRIU may translate into a change in health anxiety 6 months later. However, the occurrence and intensity of this effect seem to vary with regard to overall health anxiety level, with adolescents with comparatively high health anxiety being intact by it. Table S1 in Multimedia Appendix 1 shows the comprehensive results, including CIs. The effects that are of main interest with regard to the RQs are captured in the cross-lagged paths. The autoregressive paths capture within-person stability for each of the constructs in time. The between-person correlation reflects the stable association between the constructs across all waves.

## Discussion

### Overview

Although adolescent mental health and well-being have become central concerns, especially after the COVID-19 pandemic [44], adolescent health anxiety remains an understudied topic [2]. The pandemic may have intensified health-related concerns, leading to increased levels of acute health anxiety and greater use of health-related internet resources. According to the integrative model of health anxiety and online health information seeking [17], this may serve as a gateway to long-term health anxiety and excessive HRIU, or cyberchondria, among some adolescents [19].

This study aimed to explore the long-term within-person reciprocal effects between health anxiety and HRIU among adolescents. We built our assumptions on 2 theoretical models related to health anxiety. First, based on the integrative model [17], we hypothesized that fluctuations in health anxiety and in HRIU influence each other. The model posits that an increase in health anxiety compared to one’s own base level may prompt HRIU and, at the same time, can itself be triggered by online health information. The model suggests that such effects, although stemming from a single situation, could be detectable over time, as both successful and worrisome search may reinforce the seeking behavior over time, eventually arriving at another piece of information triggering health anxiety [17]. Second, based on the cognitive-behavioral model [15], we acknowledged that this relationship may vary across different individuals, with users having a higher base level of health anxiety being more susceptible to this vicious cycle.

Therefore, the study also uniquely examined these effects separately for adolescents with different base-level health anxiety. This approach has been adopted before, but only for adults and only distinguishing those with high level of health anxiety, showing—contrary to the cognitive-behavior model—that at a certain (ie, high) base level of health anxiety, neither health anxiety nor HRIU affects each other, likely due to a ceiling effect and a low variance [28]. We aimed to replicate this finding for the adolescent sample and expand it to those with lower base level of health anxiety. A cross-sectional study has shown that this group may not be homogeneous, suggesting that adolescents with medium and low base levels of health anxiety are different in their reaction to health-related information [13]. In line with this study, we thus focused separately on adolescents with high, medium, and low base-level health anxiety compared to others.

Our findings indicate that cross-sectionally, adolescents with higher base-level health anxiety tend to use the internet for health-related purposes more frequently, aligning with previous literature [45]. This pattern is particularly prominent in adolescents with high health anxiety, who often use health-related content as a coping mechanism and maintenance behavior [14,46]. This effect is even more apparent when comparing the group of adolescents with high health anxiety to the other 2 groups, where this effect did not appear.

However, our main objective in line with the integrative model [17] was to investigate whether health anxiety and HRIU influence each other over the long term within one individual. Notably, much of the existing research has relied on cross-sectional data [11,47], which limits the ability to explore potential bidirectional effects. To address this gap, we used an RI-CLPM to focus on within-person changes, thus providing the only longitudinal evidence on this topic for adolescents to date. This approach allowed us to determine if fluctuations in adolescents’ HRIU led to subsequent variation in their health anxiety over time, and vice versa.

### Disentangling the Long-Term Effects

#### From Health Anxiety to HRIU

The integrative model [17] assumes that health anxiety drives individuals to engage in HRIU, either to seek health-related information or to temporarily alleviate anxiety, and that this effect may be durable and prevail beyond the specific search session [18]. Although qualitative evidence supports this claim [14], it was rarely tested beyond cross-sectional findings. However, the sole longitudinal study on adults [28] found some support for this effect. Yet, contrary to the integrative model and to our first hypothesis (H1), our study failed to find a significant increase in HRIU compared to personal mean following heightened health anxiety in the ungrouped sample. This could be interpreted in various ways.

First, it is possible that although the effect proposed in the integrative model [17] should be durable beyond the search session, it may still be relatively short-term, possibly detectable within hours or days [14,20], and could not be captured in our data. Within this logic, our results suggest that although HRIU may fluctuate in response to a change in health anxiety, this effect is temporary and does not translate to increased HRIU that is still detectable after months. This would imply that HRIU may mostly play its intended role of answering momentary health concerns and calming the adolescent down. This is opposed to the maladaptive function of HRIU as suggested by the integrative model and potentially visible in the long term [17,18].

Alternatively, any possible long-term impact might have been overshadowed by external factors, such as the COVID-19 pandemic, which has significantly influenced both anxiety levels [48] and online engagement with health-related information [49]. However, this would again downplay the effect of fluctuations in health anxiety on long-term change in HRIU compared to other potential factors.

Finally, concerning the cognitive-behavioral model of health anxiety, it is possible that the effect hypothesized in H1 may still be observable in the long term but may differ for adolescents with different base-level health anxiety in our sample, which may confound the results in the ungrouped model. To acknowledge that, we looked into the groups of adolescents with high, medium, and low levels of health anxiety compared to others separately, which we discuss below in the More Complexity: Different Effects for Different Adolescents section.

#### From HRIU to Health Anxiety

The integrative model also posits that although HRIU should alleviate health concerns by providing more insight and explanations [50], it can paradoxically trigger or reinforce health anxiety [17,20]. However, while most studies have focused on this effect in the short term [20,26], te Poel et al [28] showed that it may still be detectable after 2 months, potentially posing a threat to long-term well-being. Our study aimed to explore this effect in adolescents and test whether it could still be detectable after a substantially longer lag, specifically 6 months. In line with our expectations (H2), a change in HRIU compared to one’s personal mean translated into a change in health anxiety 6 months later, though this effect was only observed between Wave 2 and Wave 3. Such a result supports the assumption that the effect of HRIU on health anxiety may be lasting.

However, our results need to be interpreted with regard to the COVID-19 pandemic. First, we are unable to distinguish the effect of health-related information specifically related to the pandemic, and we cannot predict that the same effect would be observed outside of the pandemic. For instance, during the later waves of our study, the internet saw polarized and confounded debate about COVID-19 vaccines, which, at the same time, presented an endorsed health-related topic [51]. Therefore, future studies should focus on replicating this effect in a more stable and pandemic-free context.

We have mentioned that the course of the pandemic may present a confounder that is difficult to account for. However, we believe that several of its features are more stable and should be considered when interpreting the results. Due to health topics being omnipresent, we can expect both HRIU and health anxiety to be elevated even in adolescents with lower health anxiety [19,49,52]. Additionally, a health crisis will increase the likelihood of experiences that can trigger health anxiety in adolescents in general [7].

Through this lens, the nonsignificant effects from health anxiety on HRIU between Waves 1 and 2 may be due to the ambivalent pandemic context in Wave 1 (see Methods section and [33]). While many adolescents would be relieved by the improving situation, others would be distressed by the recent culmination with strict restrictions and near collapse of the health care system, potentially leading to infection or death in their families shortly before the data collection. Such circumstances are independent of base-level health anxiety and may have led to unpredictable variability in HRIU and health anxiety. In contrast, Waves 2 and 3 likely had a more uniform impact on both variables.

On the other hand, the pandemic context in Waves 2 and 3 may underline the significance of our findings. We have described in the Methods section that the pandemic situation in Wave 2 was serious and the restrictions and number of infections were on the rise, while in Wave 3, it was calm and presented a factual restoration of the prepandemic state in terms of health care and policy. As a result, most of the pandemic-related stressors that might contribute to health anxiety should diminish from Wave 2 to Wave 3, supporting the claim that an increase in health anxiety in Wave 3 was a result of a change in HRIU in Wave 2, rather than of the acute pandemic situation.

### More Complexity: Different Effects for Different Adolescents

#### Overview

According to the cognitive-behavioral model of health anxiety [15], people with higher base level of health anxiety are more likely to have more catastrophic cognitions, seek health information for reassurance, and be more intolerant of uncertainty. Experimental studies have supported this claim, showing that the base level of health anxiety influences both HRIU and individuals’ responses to such content, making users with higher health anxiety linearly more susceptible to the vicious cycle of health anxiety and HRIU in the short term [21,24]. Nonetheless, the scarce longitudinal evidence showed that for adults with high base level of health anxiety, fluctuations in health anxiety and in HRIU do not affect each other, and that this effect was present in those with rather lower health anxiety [28], challenging the theory in the long term. To understand this better, we tested this finding on adolescents but expanded it by focusing in more detail on those with lower base-level health anxiety.

For analytical reasons, we decided to divide the sample into 3 groups based on their base level of health anxiety. We acknowledge that this grouping is somewhat artificial, as further discussed in the Limitations section. However, the groups were designed in line with the findings from a cross-sectional study that identified some differences between adolescents with low and medium levels of health anxiety [13]. Specifically, we examined adolescents with medium and low trait health anxiety (up to the 33rd percentile) in comparison to those with high trait health anxiety (above the 66th percentile; RQ1 and RQ2).

#### High Base-Level Health Anxiety

In line with previous literature [11], adolescents with high base-level health anxiety were the most frequent users of health-related media. Yet, similar to adults [28], no relationship was observed between fluctuations in their health anxiety and their HRIU. This finding has implications for both theoretical models. According to the cognitive-behavioral model, adolescents with high base-level health anxiety differ in cognitions and search strategies, which have been shown to lead to more disturbing and endless search in the short term [24,27]. As a result, they should be the most susceptible to the vicious cycle outlined in the integrative mode. However, we show that despite their frequent use, health-related media do not seem to contribute to higher health anxiety for these adolescents in the long term.

Contrary to the prevailing beliefs [16], te Poel et al [28] argue that while HRIU has a potential to fuel health anxiety, for users with high base-level health anxiety, it serves as a maintenance behavior related to health anxiety, rather than its cause. Analytically, this may translate into a ceiling effect, that is, a state in which both health anxiety and HRIU are persistently too high to vary. Our study indicates that this ceiling effect is already present in younger populations. With regard to the integrative model [17], HRIU of adolescents with high base-level health anxiety rarely leads to change in health anxiety in any way, as suggested in the initial stages of the model. Instead, it likely represents the continuous, problematic online health research proposed in the later stage of the model.

Future research should focus on disentangling problematic online health research in this subgroup to understand whether the initial stages of the model could have been observable earlier or whether the mechanism of developing problematic online health research may be different for adolescents. Altogether, for adolescents with high base level of health anxiety, HRIU will likely not increase health anxiety over time. At the same time, although they like to use it for reassurance, it will likely not decrease their health anxiety either, highlighting the need for alternative support and interventions.

#### Medium Base-Level Health Anxiety

Our focus on adolescents with medium base-level health anxiety aligns with the limited existing literature on this subgroup. Te Poel et al [28] show that people with lower, rather than higher, base levels of health anxiety are susceptible to the vicious cycle of health anxiety and HRIU, which seems to go against the cognitive-behavioral model of health anxiety. Yet, the susceptibility to this cycle may increase with higher base-level health anxiety, as the theory suggests, but this growth is not infinite, as observed in adolescents with high base-level health anxiety.

Aligning with our expectations and the existing literature [13,28], for adolescents with medium trait health anxiety, increased HRIU was consistently associated with higher health anxiety across all waves. Reciprocal effects between HRIU and health anxiety were observed between Waves 2 and 3, indicating that adolescents with medium trait health anxiety may be particularly vulnerable to the negative effects of health-related media use [46]. Such findings resemble the initial pattern outlined by the integrative model [17], suggesting that this subgroup may be prone to being triggered by HRIU, leading to both higher health anxiety and HRIU persisting over months.

This finding is significant because this subgroup has often been studied alongside people with high health anxiety and treated as a control and homogeneous group [20,28]. Yet, a recent study has shown that adolescents with medium base level of health anxiety resemble those with high health anxiety in their reaction to HRIU [13], making them prone to the adverse effects. Our results highlight that adolescents with medium trait health anxiety should be studied separately from both high and low trait health anxiety groups and be considered as a susceptible population. Likely, they have not yet developed high health anxiety or problematic online health research but may already exhibit maladaptive cognitive patterns proposed in the cognitive-behavioral model, such as intolerance of uncertainty and catastrophic thinking [24,27], making their HRIU riskier.

However, in considering these findings, we cannot rule out possible contextual factors. Given the varied nature of the pandemic in Wave 1, it is barely possible to deduce how the HRIU at this time may have looked like. Therefore, future research should focus on this at-risk but underexplored group in a postpandemic context, examining the specific patterns and goals of HRIU and cognitive mechanisms.

#### Low Base-Level Health Anxiety

The differentiation between adolescents with medium and low trait health anxiety also revealed a unique pattern for those with low trait health anxiety. On a between-person level, these adolescents engage less frequently with health-related content compared to their peers. Yet, in our study, HRIU in Wave 1 led to increased health anxiety in Wave 2, which then spurred more HRIU in Wave 3. This reciprocal relationship resembles that of adolescents with medium base-level health anxiety, but with no continued effects of HRIU on health anxiety between Waves 2 and 3.

This suggests that adolescents with low trait health anxiety might be susceptible to risky HRIU but, at the same time, more resilient to the cycle of health anxiety and HRIU [25,52]. In line with the integrative model, they may be more likely to terminate the search, thus breaking the vicious cycle [17]. The two potential mechanisms are likely. First, having lower health-related worries, they may use the internet in a less high-stakes way, potentially being less triggered by health-related information and seeking information for other purposes than reassurance. In line with the model, this may lead to a quick termination of the search. Second, it is likely that while their health-related content may trigger health worries, which then reinforces the search behavior, this group is more likely to use coping strategies that help return their anxiety and internet use to baseline levels over time.

To this date, users with low base-level health anxiety of any age have not been given attention. While the maladaptive cognitions in people with high health anxiety are well-studied, it is unclear whether there may be any cognitive strategies that serve as a protection and could be detectable in this subgroup. If so, future research should explore strategies that prevent compulsive or distressing HRIU, as they could inform treatment for adolescents with higher trait health anxiety. Moreover, as this subgroup is the least interested in health content on the internet and should be generally the least prone to health-related worries, both were nearly inevitable during the COVID-19 pandemic. As a result, we can expect that, especially for this subgroup, HRIU or health anxiety may not be representative of their usual feelings and behavior. Therefore, our findings need to be tested outside pandemic times.

### Limitations

Several limitations should be considered when interpreting our results. First, our data do not capture respondents’ health anxiety and HRIU before or long after the pandemic, making it unclear whether the observed effects reflect a broader long-term pattern influenced by preexisting tendencies. Additionally, the longitudinal design cannot explain mechanisms occurring between waves or effects of third variables, such as learning about a disease in the family, which could simultaneously impact both variables. Future research should use methods like repeated ecological momentary assessment to examine these effects in the short term while maintaining a longitudinal perspective. Such a solution could shed light on the nonsignificant paths and show whether they are truly nonexistent or simply too short-lived, as we discussed. Additionally, the results may be influenced by the 6-month time lag, which helps identify effects that remain stable across seasons and pandemic waves. However, this time lag does not allow for a clear interpretation of the nonsignificant paths. Shorter time lags could provide additional insight into whether these effects are truly nonexistent. Ideally, such studies should use lags of less than 2 months, as similar nonsignificant effects could be observed already within this time frame [28].

Second, the multigroup results should be interpreted with caution. Due to the study’s inclusion in a broader project, participants were not sampled based on health anxiety, resulting in unequal group representation. Moreover, grouping was determined empirically (based on percentiles), as the scale lacked specific cutoff points for high or clinical health anxiety. Additionally, previous research has not differentiated within the lower health anxiety subsample, leaving no established guidelines. Therefore, our grouping highlights contrasts rather than defining clear cutoffs, and alternative approaches could yield different results. For instance, defining a no-anxiety subgroup instead of a low-anxiety one might sharpen contrasts. Future research should establish reliable cutoff criteria for study and counseling use. Studies should identify whether this can be distinguished solely by the level of health anxiety or rather from a checklist of cognitions and behaviors related to this level.

Finally, the study was conducted during and after the COVID-19 pandemic, likely influencing both variables in ways that are difficult to quantify. While the context in certain waves may have highlighted our findings, others may have introduced confounding factors. Given the evolving nature of the pandemic, controlling for these influences was nearly impossible. Either way, the pandemic’s impact on health anxiety and media use may not fully reflect typical adolescent behavior, making it challenging to isolate its effects [13,33]. Additionally, although similar patterns have been observed outside a pandemic [28], it remains unclear whether these changes persist post pandemic. Therefore, despite providing valuable insight into adolescent health anxiety and HRIU during and after the pandemic, future research should retest our findings to determine which effects stem from the pandemic context and which may be expected regardless of it.

### Conclusions

To date, the causal long-term interaction between health anxiety and HRIU in adolescence has been unclear. Our study offers new insights into this long-term dynamic and into adolescent health anxiety, which, unlike in the adult population, is still relatively neglected and understudied [6].

In line with longitudinal findings on adults [28], we show that while adolescents with high trait health anxiety frequently use health-related media, it is those with low and moderate health anxiety who show increased health anxiety in response to heightened HRIU, and vice versa. For adolescents with high base-level health anxiety, frequent HRIU does not necessarily lead to adverse effects but also does not reduce health anxiety. Practitioners should acknowledge this, and while they do not have to discourage HRIU in clients with health anxiety, they should inform them that it is not efficient to reduce it as they may believe.

On the other hand, adolescents with medium base-level health anxiety were newly identified as particularly vulnerable to the negative effects of HRIU. To prevent developing higher health anxiety, this group should be guided toward higher eHealth literacy and healthy coping mechanisms. The focus on eHealth literacy (ie, critical thinking related to health information on the internet) should foster the ability to recognize that online information is often general and may not apply to individual cases or that the media often tend toward sensationalism. Coping mechanisms should prevent search escalation and compulsive HRIU as a source of relief, for instance, by setting a time limit for the search or not searching for health-related information when distressed or unable to fall asleep. Moreover, future research should focus on defining this population more precisely, especially refining the cutoff between this group and adolescents with low base-level health anxiety. Additionally, we recommend focusing on health-related habits and cognitions of the previously overlooked group with low health anxiety. Further investigation into their coping strategies could provide valuable insights for developing interventions to break the cycle of health anxiety and HRIU.

To conclude, this study provides the first insight into the bidirectional relationship between adolescent health anxiety and their HRIU. We have shown that these effects are relevant for adolescents and can be detected over as long as 6 months. Moreover, grounded in 2 theoretical models, our study both supports their validity and highlights certain limitations. Consistent with the integrative model by Brown et al [17], we show that the vicious cycle between health anxiety and HRIU operates regardless of initial health anxiety levels. However, contrary to Brown et al [17] and in line with the cognitive-behavioral model [15], we demonstrate that these effects are influenced by the base level of health anxiety. Similar to te Poel et al [28], our findings indicate that the relationship may not be linear, with the highest base-level health anxiety being less predictive than commonly expected. These findings highlight the critical role of individual differences in adolescent health anxiety, guiding future research and interventions targeting health-related behaviors.

## Appendix

Multimedia Appendix 1 Results of the random intercept cross-lagged panel model of longitudinal within-person interactions between health anxiety and health-related internet use in adolescents over 6 months, with and without grouping.

## Abbreviations

CFI: comparative fit index
H1: hypothesis 1
H2: hypothesis 2
HRIU: health-related internet use
RI-CLPM: random intercept cross-lagged panel model
RMSEA: root-mean-square error of approximation
RQ: research question
SRMR: standardized root-mean-square residual
TLI: Tucker-Lewis index

## Data Availability

The data and analysis scripts are available through the Open Science Framework [43].
