# Supplementary material for: Relationship Between Adolescent Health Anxiety and Health-Related Internet Use: 3-Wave Longitudinal Survey Study
Source: J Med Internet Res. 2025 Oct 14;27:e66129. doi: 10.2196/66129 (PMC12569483; doi:10.2196/66129)
Supplement: Multimedia Appendix 1 [file jmir_v27i1e66129_app1.docx]

**Table S1.** Results of the random intercept cross-lagged panel model (RI-CLPM) testing the longitudinal within-person interactions between health anxiety and health-related internet use in Czech adolescents aged 11-16 years at baseline (3-wave study with 6-month intervals; N=2500; data collected from June 2021 to June 2022), presented with and without grouping**.** Notes*:* HA=Health anxiety; HRIU=HRIU; STDYX standardized results; significant effects are in bold. Note that residual correlations reflect within-person associations after adjusting for model effects and do not represent simple within-wave correlations. For such an effect, see the between-person correlation.

|  | **Ungrouped** | | | | **High HA** | | | | **Medium HA** | | | | **Low HA** | | | |
| --- | --- | --- | --- | --- | --- | --- | --- | --- | --- | --- | --- | --- | --- | --- | --- | --- |
|  | β | 95% CI | SE β | *P* | β | 95% CI | SE β | *P* | β | 95% CI | SE β | *P* | β | 95% CI | SE β | *P* |
| **Autoregressive Effects** |  |  |  |  |  |  |  |  |  |  |  |  |  |  |  |  |
| W1_HA → W2_HA |  |  |  |  |  |  |  |  |  |  |  |  |  |  |  |  |
|  | .07 | -.05; .19 | .06 | *.26* | -.06 | -.33; .22 | .14 | *.70* | .03 | -.30; .35 | .16 | *.88* | **.19** | .05; .33 | .07 | *<.001* |
| W2_HA → W3_HA |  |  |  |  |  |  |  |  |  |  |  |  |  |  |  |  |
|  | **.13** | .02; .24 | .06 | *.02* | **.27** | .16; .38 | .06 | *<.001* | **.36** | .24; .47 | .06 | *<.001* | **.47** | .39; .55 | .04 | *<.001* |
| W1_HRIU → W2_HRIU |  |  |  |  |  |  |  |  |  |  |  |  |  |  |  |  |
|  | **.19** | .09; .30 | .05 | *<.001* | **.18** | .01; .35 | .09 | *.04* | **.27** | .03; .50 | .12 | *.03* | **.22** | .07; .37 | .08 | *.003* |
| W2_HRIU → W3_HRIU |  |  |  |  |  |  |  |  |  |  |  |  |  |  |  |  |
|  | **.31** | .22; .41 | .05 | *<.001* | **.24** | .07; .40 | .09 | *.01* | **.36** | .18; .55 | .09 | *<.001* | **.40** | .28; .52 | .06 | *<.001* |
| **Cross-lagged Effects** |  |  |  |  |  |  |  |  |  |  |  |  |  |  |  |  |
| W1_HA → W2_HRIU |  |  |  |  |  |  |  |  |  |  |  |  |  |  |  |  |
|  | -.01 | -.10; .10 | .05 | *.88* | -.12 | -.33; .01 | .11 | *.26* | -.19 | -.56; .18 | .19 | *.31* | .06 | -.08; .20 | .07 | *.41* |
| W2_HA → W3_HRIU |  |  |  |  |  |  |  |  |  |  |  |  |  |  |  |  |
|  | .04 | .05; .12 | .04 | *.41* | .12 | -.01; .24 | .07 | *.07* | **.15** | .01; .30 | .07 | *.03* | **.11** | .01; .21 | .05 | *.03* |
| W1_HRIU → W2_HA |  |  |  |  |  |  |  |  |  |  |  |  |  |  |  |  |
|  | -.01 | -.11; .09 | .05 | *.89* | -.01 | -.16; .14 | .08 | *.90* | **.16** | .02; .31 | .07 | *.03* | **.17** | .08; .27 | .05 | *<.001* |
| W2_HRIU → W3_HA |  |  |  |  |  |  |  |  |  |  |  |  |  |  |  |  |
|  | **.10** | .00; .20 | .05 | *.048* | .11 | -.02; .24 | .07 | *.09* | **.18** | .04; .32 | .07 | *.01* | .09 | .00; .18 | .05 | *.07* |
| **Covariances** |  |  |  |  |  |  |  |  |  |  |  |  |  |  |  |  |
| Correlation W1 |  |  |  |  |  |  |  |  |  |  |  |  |  |  |  |  |
|  | **.11** | .01; 20 | .05 | *.03* | -.06 | -.28; .17 | .12 | *.63* | -.14 | -.56; .28 | .21 | *.51* | .16 | -.03; .34 | .09 | *.10* |
| Residual correlation W2 |  |  |  |  |  |  |  |  |  |  |  |  |  |  |  |  |
|  | **.13** | .03; .22 | .05 | *.01* | **.14** | .01; .28 | .07 | *.04* | **.22** | .09; .35 | .07 | *.001* | **.24** | .15; .32 | .04 | *<.001* |
| Residual correlation W3 |  |  |  |  |  |  |  |  |  |  |  |  |  |  |  |  |
|  | **.23** | .15; .31 | .04 | *<.001* | **.32** | .21; .43 | .06 | *<.001* | **.22** | .09; 36 | .07 | *.002* | **.21** | .12; .30 | .05 | *<.001* |
| **Between-person corr.** |  |  |  |  |  |  |  |  |  |  |  |  |  |  |  |  |
|  | **.52** | .43; .60 | .04 | *<.001* | **.18** | .00; .36 | .09 | *.048* | .38 | -.02; .77 | .20 | *.07* | .12 | -.39; .63 | .26 | *.65* |

*Notes:* HA = Health anxiety; HRIU = Health-related internet use; STDYX standardized results; significant effects are in bold
